# Glasses for bone regeneration: structural features controlling physical properties and ion release of bioactive glasses 45S5, S53P4 and 13-93

**DOI:** 10.1039/d4ra06081d

**Published:** 2025-02-14

**Authors:** Zhaorui Jin, Daniel R. Neuville, Delia S. Brauer

**Affiliations:** a Otto Schott Institute of Materials Research, Friedrich Schiller University Lessingstr. 12 (AWZ) 07743 Jena Germany delia.brauer@uni-jena.de; b Géomateriaux, Institut de Physique du Globe de Paris 1 rue Jussieu 75005 Paris France

## Abstract

The structure, *i.e.* atomic arrangement, of glasses is known to determine many of their properties. This study investigates the structure of three well-known bioactive glass compositions, 45S5 (known as Bioglass), S53P4 (commercialised as BonAlive) and 13-93 (developed for improved high-temperature processing) by Si-29 and P-31 solid-state nuclear magnetic resonance and Raman spectroscopy. Results show that 45S5 has a more depolymerised silicate structure than the other two glasses, in agreement with its lowest silica content. These structural differences explain the well known high solubility and fast reactivity *in vivo* of 45S5 compared to the other two compositions. Differences between S53P4 and 13-93, by contrast, originate more from differences in their average modifier field strength, as their network connectivity, *i.e.* average silicate network polymerisation, is similar. As a result, 13-93 shows the lowest crystallisation tendency of the three glasses but also reacts relatively slowly during contact with aqueous solutions. The structural differences are also reflected in glass viscosity, where at a given temperature 45S5 has the lowest viscosity, 13-93 the highest and S53P4's viscosity is lying in between.

## Abstract 2

This study investigates the atomic structure of three well-known bioactive glasses and how they affect common glass properties.

## Graphical abstract

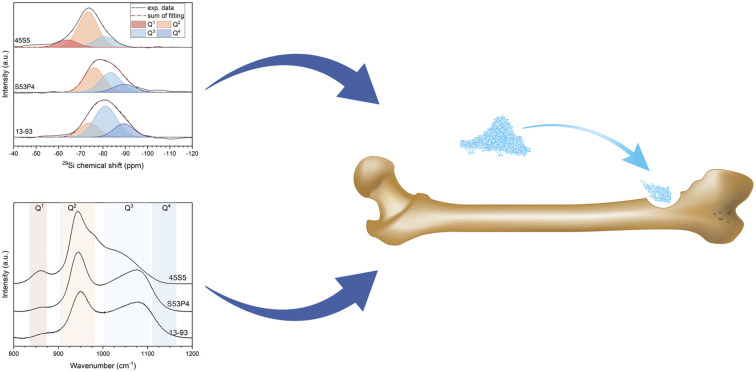

## Introduction

The first bioactive glass, composition 45S5 (often referred to as “Bioglass”), was developed in the late 1960's, has been in clinical use since the mid-1980s and is used successfully to regenerate bone.^1^ Another bioactive glass used clinically, S53P4 (commercialised as BonAlive), is used to treat chronic bone infections (osteomyelitis).^2^ As bioactive glasses typically devitrify easily at elevated temperatures, composition 13-93 was developed as a bioactive glass with improved processing,^3^ and to this day it is the composition of choice for the preparation of sintered porous scaffolds.^4,5^

It is known that the atomic (or molecular) arrangement in glasses determines their properties. As a result, structural analyses, especially by solid-state nuclear magnetic resonance (NMR) spectroscopy techniques, are now commonly performed on glasses to elucidate how their atomic set-up is related to their macroscopic properties. Computer simulations have identified structural features directly involved in the interaction between bioactive silicate glasses and water,^6–9^ and while some of these features, especially the ones present at the glass surface only,^6–8^ cannot currently be detected by solid-state NMR or Raman spectroscopy, certain features in the bulk can be detected and quantified.

Several studies have investigated the structure of Bioglass 45S5.^10–13^ However, to the best of our knowledge, to date no detailed structural studies have been published on the well-known bioactive glasses S53P4 and 13-93. Here we investigate how the molecular structure of these three well-known bioactive glasses differs, and how this affects and controls their physical properties. We further correlate their structure with published data of the glasses' behaviour during contact with physiological fluids.

## Results and discussion

### Structural analysis

**Fig. 1:**
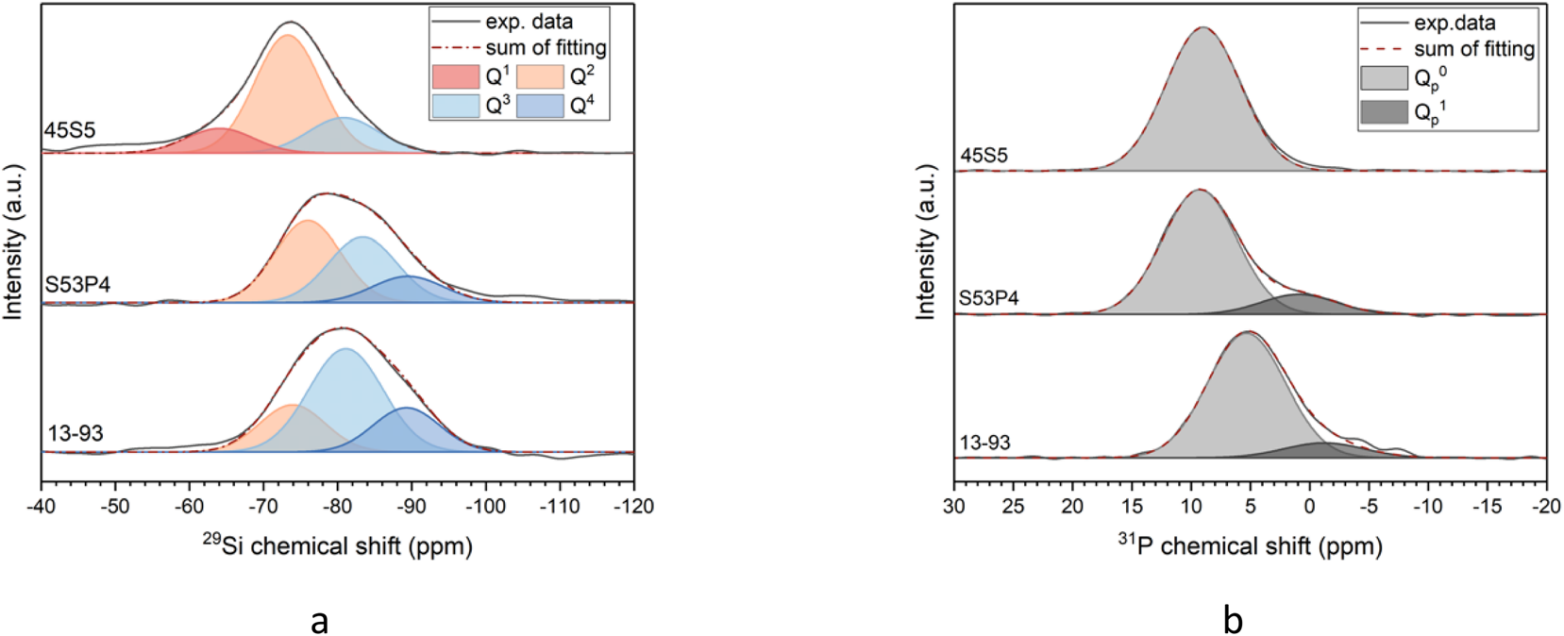
(a) ^29^Si and (b) ^31^P MAS NMR spectra and their deconvolution for glasses 45S5, S53P4 and 13-93.

^29^Si MAS NMR spectra of the glasses (Fig. 1a) exhibit a single broad signal between −50 and −105 ppm. The maximum position of glasses S53P4 and 13-93 is positioned at more negative ppm values compared to 45S5. This indicates that the former two glasses are more polymerised,^14^ owing to their higher silica and lower modifier oxide contents.

In silicate glasses, the main building units of the network are SiO_4_ tetrahedra of silicon atoms surrounded by four oxygen atoms. These tetrahedra are usually referred to as Q^*n*^ groups, with *n* being the number of bridging oxygen atoms (≡Si–O–Si≡) connected to the silicon atom. The deconvolution of the ^29^Si MAS NMR spectra into Gaussian signals for the different Q^*n*^ species provides information on the relative amounts of Q^*n*^ groups (Fig. 1a). Results agree with the differences in silicate network polymerisation expected for different silica contents. The position of the peak maximum, line width and relative amounts of Q groups are presented in Table 1.

**Table 1:** Observed ^29^Si (Q^*n*^) and ^31^P MAS NMR (Q_P_^*n*^) peak position (*δ*), linewidth (full width at half maximum, both in ppm), relative amounts of Q groups based on MAS NMR results (*I* in %) and nominal composition (*I*_th_; assuming a binary distribution) and network connectivity (NC) based on the nominal composition (assuming a binary distribution) and on ^29^Si MAS NMR results

		45S5	S53P4	13-93
Q^1^	*δ* (fwhm)	−64.1 (10.7)	—	—
	*I*	14	—	—
	*I*_th_	0	0	0
Q^2^	*δ* (fwhm)	−73.2 (10.5)	−76.0 (10.5)	−73.9 (10.4)
	*I*	65.1	46.1	22.6
	*I*_th_	88	46	41
Q^3^	*δ* (fwhm)	−80.8 (11.1)	−83.4 (10.9)	−80.1 (11.8)
	*I*	20.9	38.0	55.9
	*I*_th_	12	54	59
Q^4^	*δ* (fwhm)	—	−89.5 (11.2)	−89.3 (10.5)
	*I*	—	15.9	21.5
	*I*_th_	0	0	0
Q^0^_P_	*δ* (fwhm)	9.0 (7.4)	9.3 (7.4)	5.3 (7.7)
	*I*	100	86	89
	*I*_th_	100	100	100
Q_P_^1^	*δ* (fwhm)	—	0.9 (7.5)	−1.2 (8.0)
	*I*	—	14	11
	*I*_th_	—	0	0
NC	Theor.	2.12	2.54	2.59
	^29^Si NMR	2.07	2.70	2.99

In 1D ^29^Si MAS NMR spectra of silicate glasses, the peaks of different Q groups overlap, adding an arbitrary element to the fitting without any additional constraints. As a result, data in the literature have been fitted using either two or three signals, corresponding to either Q^2^ and Q^3^ groups only or Q^1^, Q^2^ and Q^3^.^10–13,15^ Pedone *et al.* combined their structural analysis by solid-state NMR methods with computer simulations and concluded that a trinomial distribution of Q groups, *i.e.* including Q^1^ to Q^3^, provides the best agreement between the two methods.^10^ In the present study, we also opted for fitting using three peaks. Results for all three glasses are shown in Fig. 1a and in Table 1.

Glass 45S5 shows mostly Q^2^ species (*i.e.* chain middle groups, Fig. 1a), with small amounts of Q^3^ (branching units) and Q^1^ (chain end groups), in good agreement with the relative amounts found by Pedone *et al.*^10^ The structure of 45S5 therefore is a weakly cross-linked 3D network, containing significant amounts of chain-like fragments in the silicate network. By contrast, we could not detect significant amounts of Q^1^ for glasses S53P4 and 13-93. For S53P4, the largest contribution still originates from Q^2^ groups (46.1%), followed closely by Q^3^ at 38%. S53P4 shows the presence of Q^4^ groups (*i.e.* units branching into four directions) as well. The structure of glass 13-93 is dominated by Q^3^ species, with Q^4^ and Q^2^ being present to a lesser extent, corresponding to a 3D silicate network.

Owing to the low natural abundance of the ^29^Si isotope, ^29^Si MAS NMR measurements on samples not enriched in ^29^Si, as in the present study, show much worse signal-to-noise ratios than measurements on samples enriched in ^29^Si, as in the study performed by Pedone *et al.*^10^ The resulting noise in the signal makes it more challenging to correctly identify the background signal in the spectra, as seen to the left and the right of the peaks shown in Fig. 1a. This explains why our peak positions differ slightly from those found by Pedone *et al.* Nonetheless, our relative amounts of the different Q groups for 45S5 agree very well with those found by Pedone *et al.*^10^

When looking at the composition in weight percentages (*cf.* Experimental section) which is traditionally given for bioactive glasses, we notice that S53P4 and 13-93 have the same network former contents (silica and phosphorus pentoxide) of 53% and 4%, respectively. Still, our structural analysis shows pronounced differences. This highlights the importance of considering molar (or atomic) composition in glass science rather than weight-based compositions, an aspect that has been emphasised previously.^16,17^ The composition in molar percentages reveals differences not only between 45S5 and the other two glasses but also between the silica contents of S53P4 and 13-93: 45S5 46.1 SiO_2_, 2.6 P_2_O_5_, 26.9 CaO, 24.4 Na_2_O, S53P4 53.8 SiO_2_, 1.7 P_2_O_5_, 21.8 CaO, 22.7 Na_2_O and 13-93 54.6 SiO_2_, 1.7 P_2_O_5_, 22.1 CaO, 7.7 MgO, 6 Na_2_O, 7.9 K_2_O (all in mol%).

For describing the degree of polymerisation of the silicate network, a parameter called “network connectivity” (NC) is typically used.^18^ It describes the average number of bridging oxygens per silicon atom, thereby providing an average value for *n* of all Q^*n*^ groups present in the glass. A lower NC therefore corresponds to a more depolymerised silicate network, with an NC of 2 describing a chain structure, held together by chain entanglements and ionic bridges between chains rather than by covalent cross-links. This is in contrast to the fully polymerised network of fused silica with its NC of 4. The experimental network connectivity here has been obtained experimentally from deconvolution of ^29^Si MAS NMR experiments, *i.e.* from the Q^*n*^ distribution. Nominal NC, by contrast, has been calculated from the glass composition. The latter needs to take into account that phosphate (mostly present as orthophosphate, as discussed below) requires modifier cations for charge-balancing purposes, as explained previously.^17,18^ Results (Table 1) from ^29^Si MAS NMR and the nominal composition show the same trend of 45S5 possessing the lowest NC followed by S53P4 and 13-93. For the latter two, experimental values are much higher than nominal ones, suggesting an overestimation of Q^4^ (and possibly Q^3^), most likely owing to noise as discussed above.

^31^P MAS NMR spectra of the glasses (Fig. 1b) show one single peak each between about 20 and −10 ppm. The peak of 45S5 looks symmetrical, while the peaks of the other two glasses show clear shoulders on the right-hand side, at about −2 ppm. The spectra of 45S5 and S53P4 have their maxima at 9 and 9.2 ppm, respectively, in agreement with previous ^31^P MAS NMR studies on 45S5.^10^ This is usually interpreted as phosphorus being present as orthophosphate (Q^0^_P_) charge-balanced by modifier cations.^10,19^ The maximum of the spectrum of 13-93 is found at lower ppm values compared to 45S5 (5.1 ppm; Fig. 1b and Table 1). As orthophosphate is charge-balanced by modifier cations, the type of modifier present influences the chemical shift of the signal.^15^ Lockyer *et al.* showed that a bioactive-type phosphosilicate glass containing sodium only but no calcium gives a signal at about 15.6 ppm, with the peak maximum shifting to lower ppm values as the amount of calcium increases.^19^ 13-93 has a much lower alkali metal oxide content and a higher calcium oxide content than either 45S5 and S53P4, and this causes the differences in signal position.^20,21^ Even the small difference in the chemical shift of the fitted peak for Q^0^_P_ observed for 45S5 (9.0 ppm) and S53P5 (9.3 ppm) may be caused by the former's CaO/Na_2_O ratio being slightly larger than that of the latter (1.10 for 45S5 compared to 0.96 for S53P4). Indeed, we have previously shown that, at least for sodium and calcium being the only modifier cations, the ratio of these two cations around orthophosphate reflects the overall ratio of the cations in the glass.^15^

The ^31^P MAS NMR spectra of glasses S53P4 and 13-93 show a shoulder at about −2 ppm. Mathew *et al.* detected such shoulders in melt-derived SiO_2_–P_2_O_5_–CaO–Na_2_O glasses with higher network connectivity (2.5 and 2.9) including two compositions not identical but similar to S53P5, but not for a network connectivity of 2.1. They interpreted the signal as Q_P_^1^ groups, *i.e.* phosphorus surrounded by 4 oxygen atoms, one of which being a bridging oxygen, with Q_P_^1^ making up about 10% of phosphorus,^22^ compared to 14% (S53P4) and 11% (13-93) in the present study. In a recent ^31^P MAS NMR study on a composition similar but not identical to 13-93 (referred to as “13-93-based”) the authors found slightly different peak positions but their deconvolution results were nearly identical to ours (12% Q_P_^1^).^23^ While theoretically this bridging oxygen could be connected to either another phosphorus or to silicon, Mathew *et al.* interpreted these Q_P_^1^ groups as Si–O–P bonds, based on molecular dynamics studies^24^ and experimental NMR results.^25^ We may therefore conclude that in the present study, the two glasses with higher network connectivity, S53P4 and 13-93, while mostly containing phosphorus as orthophosphate, also contain small amounts of phosphorus covalently bonded to the silicate network.

**Fig. 2:**
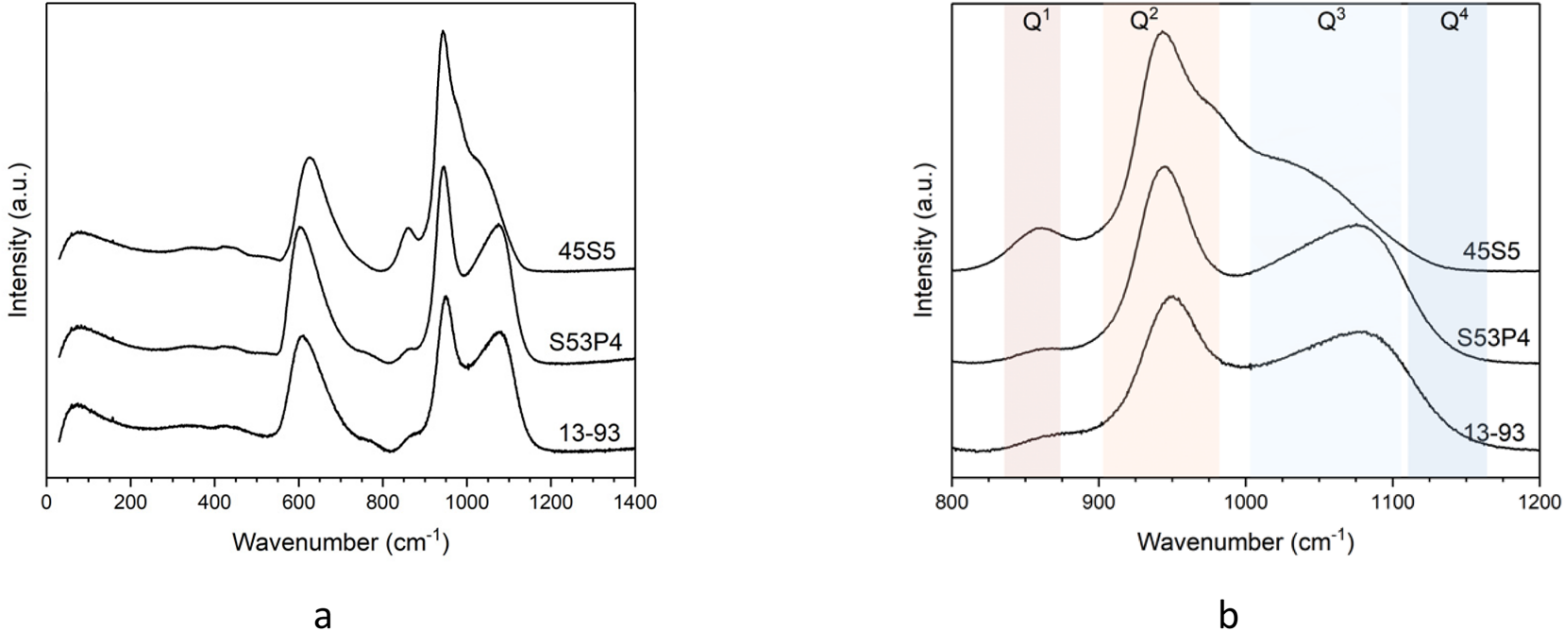
Raman spectra of glasses 45S5, S53P4 and 13-93: (a) entire spectra and (b) high-frequency region only.

Raman spectra in the range from 20 to 1400 cm^−1^ are displayed in Fig. 2. Four zones can be identified in the spectra: the Boson region from 10 to 250 cm^−1^, the low frequency region from 250 to 700 cm^−1^, the intermediate frequency region from 700 to 850 cm^−1^ and the high frequency region from 850 to 1300 cm^−1^.^26–28^ The Boson peak has been linked to rotational motions of SiO_4_ tetrahedra in vitreous silica, where the local structure is relatively well defined.^29^

Results here do not show pronounced differences in the Boson peak region of the three glasses. The low frequency region provides information on the symmetric stretching modes of Si–O–Si. The signal in this region is found at a higher frequency for 45S5 than for the other two glasses (maximum at 600 for S53P4 and 13-93 and 650 cm^−1^ for 45S5), caused by its lower silica and higher modifier oxide content.^27^ In the intermediate frequency region, a broad band at around 780 cm^−1^ has been attributed to oxygen motions in the Si–O–Si plane caused by Si–O stretching. This band has been reported to become stronger with increasing silica content,^30^ which is probably the reason why no band in this region is found for the present glasses with their low silica content.

The high frequency region includes the Si–O stretching in different Q^*n*^ tetrahedra, and this area is shown enlarged in Fig. 2b. 45S5 shows a broad band at about 860 cm^−1^, which is assigned to Si–O^−^ stretching in Q^1^ groups,^31–33^ in agreement with our ^29^Si MAS NMR results. Glasses S53P4 and 13-93 show only a very weak signal here. All three glasses show a broad band in the range from about 920 to 990 cm^−1^, corresponding to the presence of Q^2^ groups also detected by ^29^Si MAS NMR above.^31–33^ Bands in the frequency range from about 1000 to 1125 cm^−1^ correspond to Si–O^−^ stretching in tetrahedra with only one non-bridging oxygen atom (Q^3^). 45S5 shows a shoulder here only, in agreement with ^29^Si MAS NMR results. By contrast, the broad band visible for S53P4 and 13-93 confirms their more polymerised silicate structure. All three glasses show shoulders in the frequency range from 1125 to 1175 cm^−1^, which is attributed to fully polymerised tetrahedral units (Q^4^). The intensity of the shoulder of 45S5 is very low, while its intensity is higher for S53P4 and 13-93.

### Viscosity and thermal analysis

**Fig. 3:**
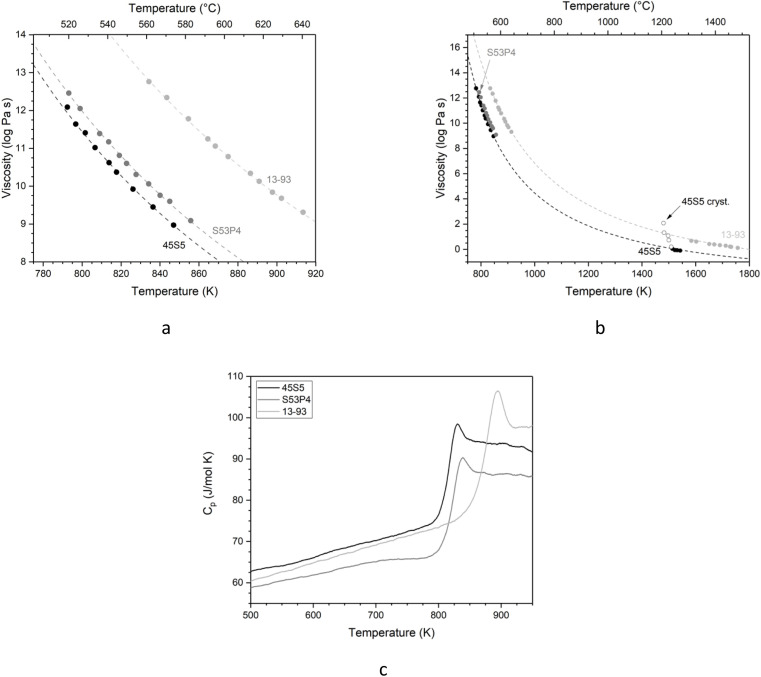
Viscosity of the glasses as a function of temperature fitted with (a) the Vogel–Fulcher–Tammann equation for measurements at low temperatures and (b) the Adam–Gibbs equation for the entire temperature range. Symbols show experimental data points and dashed lines show fitting. Open symbols represent values for 45S5 with crystallisation occurring. (c) Molar heat capacity of the glasses, plotted over the temperature.

Fig. 3a shows the low-temperature viscosity data (around *T*_g_) plotted as a function of temperature. Data were fitted using the Vogel–Fulcher–Tammann (VFT) equation (eqn (1)):^26^1
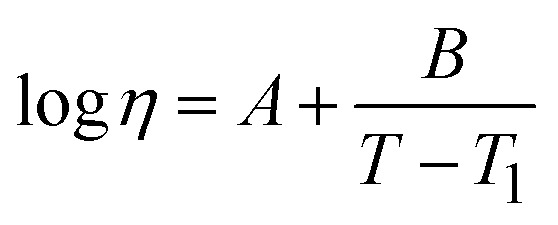


The three adjustable parameters, *A*, *B* and *T*_1_, are listed in Table 2; fitted curves are plotted in Fig. 3a. The fitting curves for all compositions are nearly parallel, representing comparable rates in viscosity change.

**Table 2:** Parameters obtained from the fitting of the low temperature viscosity data by the Vogel–Fulcher–Tammann equation (*A* in log Pa s, *B* in K(log Pa s) and *T*_1_ in K) as well as glass transition temperature obtained from viscosity measurements as the temperature corresponding to a viscosity of 10^12^ Pa s, *T*_g_(*η*), in °C

Glass	*A*	*B*	*T*_1_	*T*_g_(*η*)
45S5	−3.323	3454.8	565.9	518
S53P4	−3.872	3931.5	552.0	527
13-93	−3.963	4998.1	536.0	576

Combined data of low-temperature and high-temperature viscosity measurements were fitted using the Adam–Gibbs equation (eqn (2)):^26,34^2
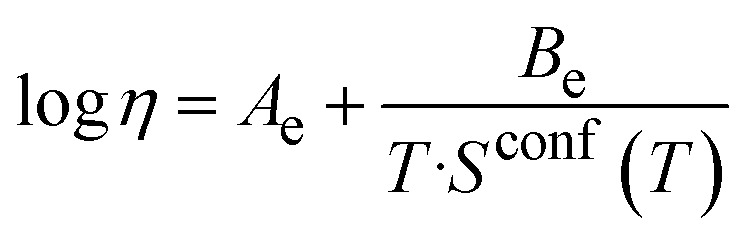
Here, *A*_e_ is a pre-exponential factor, and *B*_e_ is a parameter showing Gibbs-free energy barriers hindering configurational rearrangement in the liquid.^26^ (For glass S53P4, Adam–Gibbs fitting was calculated based on the low-temperature viscosity data and configurational heat capacity, *C*^conf^_p_ which, in turn, was calculated from heat capacity data from DSC measurements). Data obtained from fitting with the Adam–Gibbs equation are presented in Table 3; curves are shown in Fig. 3b. Eqn (2) shows that viscosity data can be linked to the configurational entropy of the melt at temperature *T*, *S*^conf^ (*T*):3
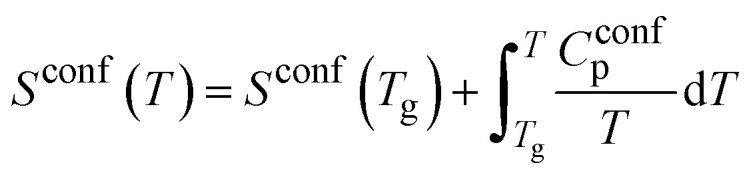


**Table 3:** Parameters obtained from the fitting of viscosity data by the Adam–Gibbs equation, *A*_e_ and *B*_e_, heat capacities of the glass at *T*_g_, heat capacity of the liquid, configurational heat capacity at *T*_g_ and the configurational entropy at *T*_g_

Glass	*A*_e_	*B*_e_	*C* ^g^_p_ (*T*_g_)	*C*_p_^l^	*C* ^conf^_p_ (*T*_g_)	*S* ^conf^ (*T*_g_)
45S5	−2.8163	88 682	73.91	93.77	19.86	7.5497
S53P4	0.91664	58 496	68.09	93.77	25.68	6.6092
13-93	−2.9904	155 400	75.58	97.80	22.22	12.194

The configurational entropy of the glass remains constant up to the glass transition temperature, *S*^conf^ (*T*_g_). Changes in configurational entropy in the melt are associated with C^conf^_p_.^26,34,35^ For silicates, *C*^conf^_p_ has been described as the difference in heat capacity between the liquid and the glass at transition temperature.^36^

Heat capacity information can also be obtained from DSC measurements, and Fig. 3c shows the molar heat capacity (*C*_p_) data for all three glasses for comparison. At low temperatures, the curves show a slight increase with increasing temperatures. At *T*_g_, the slope increases dramatically, while *C*_p_ of the melt remains constant. *C*_p_ values of the three glasses do not show pronounced differences, as all three possess highly disrupted silicate networks.

Viscosity results (Fig. 3) show that at any given temperature, 45S5 has the lowest viscosity, 13-93 the highest and S53P4 an intermediate one. This is likely caused by their differences in network connectivity observed above, with the least polymerised glass requiring the lowest temperature to reach a certain viscosity and *vice versa*. It is noticeable that the viscosity values of glasses 45S5 and S53P4 lie much closer to each other than to 13-93. This is surprising, as silica contents and network connectivity of S53P4 and 13-93 are closer to each other than to 45S5. However, besides the silicate network connectivity, modifier field strength plays a role in viscosity, in the present case the ratio of alkaline earth metal oxides (CaO + MgO) to alkali metal oxides (Na_2_O + K_2_O). As mentioned above, this ratio is 1.10 for 45S5 and 0.96 for S53P4. 13-93, by contrast, has a ratio of 2.14. And this presence of higher field strength modifier cations probably causes the higher viscosity of 13-93, together with the influence of its network connectivity.

One additional explanation may be the presence of magnesium ions in glass 13-93. Magnesium oxide is often incorporated into bioactive glasses to improve their processing,^37^ as it reduces the crystallisation tendency. Despite the larger field strength of Mg^2+^ ions compared to Ca^2+^,^38^ magnesium for calcium substitution has been shown to decrease glass transition temperature in bioactive glass ICIE1.^37^ When replacing Ca with Mg in CaSiO_3_ glass, viscosity went through a minimum for mixed compositions.^34^ Therefore, while magnesium may have an effect here, this requires further investigation.

Viscosity is closely connected with glass processing at elevated temperatures. With decreasing network connectivity, a given viscosity (as well as *T*_g_, which corresponds to a viscosity of 10^12^ Pa s) is reached at lower temperatures, making glass processing possible at lower temperatures. This advantage is counterbalanced, however, by the increase in crystallisation tendency resulting in the typical poor processing bioactive glasses of low silica content and network connectivity.^39–41^Fig. 3b includes the viscosity values for composition 45S5 once crystallisation sets in during high-temperature viscosity measurements (open symbols), showing the rapid onset of crystallisation from a certain temperature or viscosity.

**Fig. 4:**
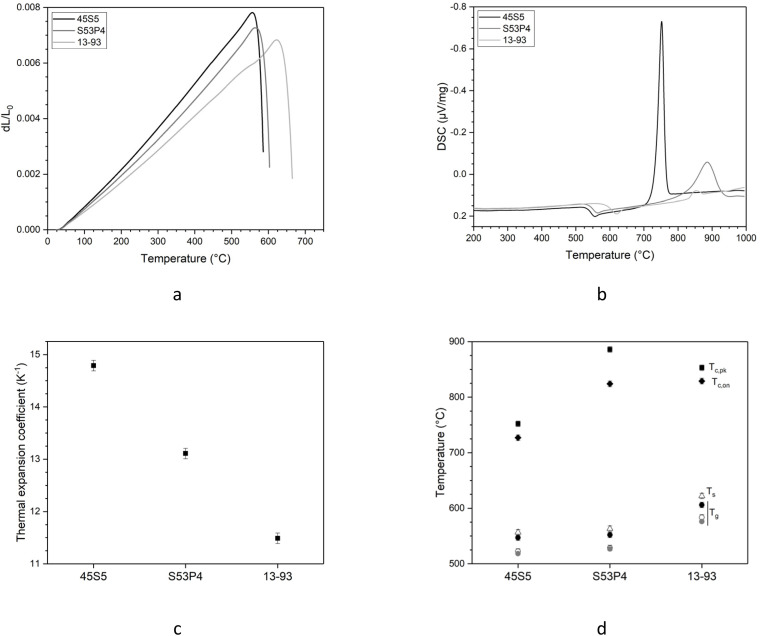
(a) Dilatometry curves, (b) DSC traces, (c) thermal expansion coefficient, *α*, obtained from dilatometry and (d) thermal results from dilatometry (open symbols) and DSC (closed symbols): glass transition temperature, *T*_g_ (circles), dilatometric softening point, *T*_s_ (triangles), as well as onset (*T*_c,on_, diamonds) and peak temperatures (*T*_c,pk_, squares) of the crystallisation exotherm. Glass transition temperature obtained from VFT fitting of viscosity measurements (*T*_g_(*η*), temperature corresponding to a viscosity of 10^12^ Pa s, grey circles) is added for comparison.

Similar to viscosity, glass transition temperature and dilatometric softening point vary with network connectivity, and as a consequence, the glasses studied here differ in these parameters, too (Fig. 4). The dilatometry curves shown in Fig. 4a clearly show differences in slope (related to the thermal expansion coefficient of the glasses, *α*) and position of maxima (representing the dilatometric softening point, *T*_s_). These two parameters, together with glass transition obtained from dilatometry, are plotted in Fig. 4c and d, showing that 45S5, with its lowest network connectivity, has the largest value for *α* and the smallest ones for *T*_g_ and *T*_s_, while 13-93 with the highest NC shows the smallest *α* and the highest values for *T*_g_ and *T*_s_. *T*_g_ values obtained from DSC and viscosity experiments (Fig. 4d) show the same trend, and this relationship with NC can be explained by a more polymerised silicate network requiring more energy to soften, agreeing with our observations from viscosity measurements above. The differences observed for the thermal expansion coefficient can also be explained by the network structure. Thermal expansion of a material is caused by the vibration of its atoms or molecules. In glasses, larger concentrations of modifier ions cause depolymerisation of the silicate network by turning bridging oxygen units into non-bridging ones and resulting in modifier cations to be coordinated by oxygen atoms (including both bridging and non-bridging ones).^10^ The vibration of the modifier cations within these oxygen polyhedra causes the thermal expansion of glasses to increase with increasing modifier concentrations.

The shape of the crystallisation peaks in DSC traces (Fig. 4b) differs for the glass compositions, with 45S5 showing a narrow, high intensity signal, S53P4 showing a broader peak of lower intensity and 13-93 showing a small signal only. The differences in crystallisation peak shape and intensity between 45S5 and S53P4 indicate differences in crystallisation mechanism in agreement with the observations by Massera *et al.*^42^ The authors analysed the crystallisation behaviour of these two glasses in detail, showing that S53P5 crystallised by a surface nucleation mechanism independent of the particle size studied. 45S5, by contrast, showed a more complex behaviour than simple nucleation and growth. The low intensity crystallisation peak of 13-93 indicates a lower crystallisation tendency of the glass, which we have shown previously.^43,44^ Fagerlund *et al.* analysed the crystallisation behaviour of 13-93 and found a surface nucleation mechanism (as in S53P4) with wollastonite (CaSiO_3_) being the primary crystal phase.^45^ Arstila *et al.* showed that the primary crystal phase of bioactive glasses gives an indication on how easily a glass can be processed at elevated temperatures without crystallisation occurring,^40^ with glasses showing wollastonite crystallisation possessing better processing than those showing crystallisation of sodium calcium silicates. This confirms that not only an increase in network connectivity reduces the crystallisation tendency but also an increase in the ratio of alkaline earth metal oxides to alkali metal oxides ratio (owing to differences in field strength between the two cations), explaining the good processing of 13-93.

The DSC traces also show differences in the temperatures at which crystallisation events are observed. The onset of the crystallisation exotherm occurs at higher temperatures for glasses S53P4 and 13-93 than for 45S5, as expected from their more polymerised silicate network and higher viscosity at a given temperature, resulting in a lower mobility of structural units to rearrange and form nuclei. This is in agreement with results published by Massera *et al.* showing that the maximum nucleation rate occurred at higher temperatures for S53P4 (608 °C) than for 45S5 (566 °C).^42^ The maximum nucleation rate reported for 13-93 occurred at even higher temperatures, 700 °C, owing to this glass's larger ratio of alkaline earth metal oxides to alkali metal oxides (and slightly higher network connectivity) than that of S53P4.^45^

### Correlation with degradation and bioactivity data from the literature

Tilocca & Cormack's molecular dynamics simulations have shown that the depolymerisation of the silicate network by modifiers plays an important role in the interaction between water molecules and glass surfaces.^6,46^ The authors showed non-crosslinked silicate chains to be present in highly bioactive compositions but absent in bio-inactive ones, which agrees with our finding that the silicate network of 45S5 is much more weakly cross-linked than that of the other two compositions and contains significant amounts of chain-like fragments. The authors further point out that less energy is required for releasing chain-like fragments into solution compared to a more cross-linked structure, and they suggest that this is likely to enable fast bioactive glass dissolution.^9^

Tilocca & Cormack also identified further surface features playing important roles in bioactive glass reactivity with water. While the experimental methods used in the present study, solid-state NMR and Raman spectroscopy, do not allow us to identify structural features present at the glass/water interface, several of the structural moieties they identified we detected experimentally. Non-bridging oxygen atoms, which solid-state NMR shows to be present in large concentrations in bioactive glasses, have been shown to act as proton acceptors, enabling spontaneous water dissociation.^7^ In addition, modifier ions act as hydrophilic sites but also as Lewis acids, and they aid in the dissociation of water molecules. The latter causes fast ion release as well as formation of Si–OH groups. As a result, Bioglass 45S5 not only releases ions faster than S53P4 or 13-93, it also forms surface layers faster, including an ion-depleted silica-rich (“silica gel”) layer and an apatite surface layer.^43,47,48^

*In vitro* immersion experiments in an acellular environment,^49^*e.g.* in simulated body fluid (SBF), Tris–HCl buffer solution or cell culture media (without addition of cells), give insight into ion release and the formation of apatite surface layers. A round robin study including glasses 45S5, S53P4 and 13-93 of the same particle size clearly demonstrated that 45S5 was the most reactive of the three glasses, both in terms of ion release (although the authors did not normalise the ions in solution to those present in the glass, making this less obvious) and in apatite precipitation, followed by S53P4. 13-93, on the other hand, was the least reactive of the three glasses and, indeed, was the only glass in the study (which included two additional compositions) which did not form apatite within 3 days.^47^ Tilocca & Cormack highlight the importance of sodium ion enrichment on the glass surface for hydrolysis reactions and the subsequent release of silicate fragments into solution.^8^ This helps to explain the differences in solubility between S53P4 and 13-93, which possess comparable silicate network connectivity but differ in their alkaline earth metal oxide/alkali metal oxide ratio, with S53P4 containing much larger sodium (*i.e.* alkali metal ion) concentrations.

In our previous study, we compared the ion release behaviour of 45S5 and 13-93 in Tris–HCl buffer solution.^43^ Our results show that the pH increase of the solution during immersion was a lot more pronounced for 45S5 than for 13-93. This is known to be directly related to the release of modifier ions,^17^ and the percentage of sodium ions released into solution was indeed higher for 45S5 than for 13-93. By contrast, the differences for calcium were less pronounced and appeared at early time points only (up to 24 hours). The reason is that calcium ions are consumed during apatite precipitation. Phosphate concentrations showed the opposite, with those for 13-93 being higher than those for 45S5, a fact also related to apatite precipitation. Both will be discussed below.

Our previous study investigated surface layer formation during immersion and showed Fourier-transformed infrared spectra of powders at 6 hours' immersion in Tris–HCl buffer to be very similar for 45S5 and 13-93.^43^ At 24 hours, however, the spectrum of 13-93 still looked like the one at 6 hours, while the one of 45S5 displayed pronounced differences: at about 1000 cm^−1^ a narrow line corresponding to calcium orthophosphate had emerged, superimposed on the bridging oxygen band. In addition, the typical split band at 560 and 600 cm^−1^, also attributed to calcium orthophosphate, was clearly visible. These features are usually taken as an indication for successful apatite formation, and it has been shown repeatedly that 45S5 may form apatite within 24 hours.^49^

This apatite formation on 45S5 explains the small difference in relative calcium concentrations and the lower phosphate concentrations compared to 13-93: As 13-93 did not precipitate any apatite, the ion concentrations in solution represent the entire amount of ions released from the glass. For 45S5, the ions in solution are those released minus the ones consumed during apatite formation. We can therefore only take sodium concentrations to indicate differences in ion release behaviour, and they confirm the statement above of the less polymerised glass 45S5 releasing ions more readily than the more polymerised glass 13-93. As 45S5 releases ions faster, it also forms surface layers faster than 13-93, owing to the higher solution pH and larger ion concentrations in solution.

At lower network connectivity, smaller silicate fragments (chains or rings) are likely to be present, as discussed above.^50,51^ Tilocca & Cormack showed small, two- or three-membered, silica rings to be present at the bioactive glass surface.^7^ While their opening is apparently impeded by an energy barrier, the authors suggested them to assist nucleation of dissolved calcium and phosphate ions at the glass surface.^7^ This indicates that in addition to faster ion release, certain bioactive glass surface features may enhance surface mineralisation in highly depolymerised silicate glasses.

Despite this influence of network connectivity, glasses may not necessarily differ that much in their *in vivo* bioactivity. A study investigating 45S5 and S53P4 showed that 45S5 formed thicker silica gel and apatite surface layers during *in vitro* immersion in simulated body fluid than S53P4 at the same time point. In addition, layers formed *in vivo* (upon implantation into soft tissue – skin or muscle – of rats) differed in some characteristics, with silica gel and apatite layers being very distinct for S53P4 but showing a gradual transition from one to the other for 45S5. However, despite these differences, total layer thickness *in vivo* was comparable for both glasses.^48^

## Experimental

### Glass synthesis

Compositions 45S5 (45 SiO_2_, 6 P_2_O_5_, 24.5 CaO, 24.5 Na_2_O), S53P4 (53 SiO_2_, 4 P_2_O_5_, 20 CaO, 23 Na_2_O) and 13-93 (53 SiO_2_, 4 P_2_O_5_, 20 CaO, 5 MgO, 6 Na_2_O, 12 K_2_O, all in wt%) were selected for this study, as they are three very well-known bioactive glasses – possibly the best known and most investigated ones. They were prepared by a melt-quench method; reagents Na_2_CO_3_ (99.5%), CaCO_3_ (98.5%), NaPO_3_ (pure), CaHPO_4_·H_2_O (98%) and SiO_2_ (99%; all Carl Roth GmbH, Germany) as well as MgCO_3_ (Sigma-Aldrich, Germany) were used as raw materials.

The raw materials were pre-dried overnight before being weighed and mixed thoroughly to make 100 g batches of glass. Glasses were melted in a covered Pt-crucible in an electrically heated furnace. To avoid overflow, the temperature was increased stepwise to the melting temperature: batches were first kept at 850 °C for half an hour for decarbonation, then the temperature was increased to 1200 °C and held there for another half hour before increasing to the respective melting temperatures. Melting temperatures and times were chosen based on the literature: 1350 °C for 1 hour for 45S5,^52^ 1360 °C for 3 hours for S53P4,^53^ and 1400 °C for 1 hour for 13-93.^54^ Then the glasses were directly quenched in water to obtain frit and dried in an oven at 100 °C overnight. Glass composition was confirmed by X-ray fluorescence analysis and the absence of crystalline phases was tested by powder X-ray diffraction (XRD; Rigaku MiniFlex 300; Cu Kα; 10 to 70° 2*θ*, 1° min^−1^; measurements performed at room temperature; results not shown).

### Thermal analysis

Dilatometry was performed on cylindric specimens (4 mm in diameter and 20 mm in length) at a heating rate of 5 K min^−1^ to obtain glass transition temperature (*T*_g_), dilatometric softening point (*T*_s_) and thermal expansion coefficient (*α*; between 100 and 400 °C).

*T*_g_ (as inflection point), onset and peak crystallisation temperatures (*T*_c,on_ and *T*_c,pk_) as well as heat capacity, *C*_p_, were measured by differential scanning calorimetry (DSC 404 F3 Pegasus®, Netzsch, Germany) using bulk samples. Samples were drilled into discs of 6 mm in diameter and 1.5 mm in height, which are similar parameters to the sapphire standard used. The side in contact with the equipment was polished with abrasive papers to grit 600 (Presi, France). After testing the background and sapphire standard, the sample was placed in a small platinum pan with lid. Experiments were performed in a platinum furnace with air supply. During *C*_p_ measurements, isothermal steps need to be considered. The sample was first increased to 40 °C with a heating rate of 10 K min^−1^ and held for 10 minutes before heating up to 1000 °C with the same heating rate. The sample remained at 1000 °C for 10 minutes for the isothermal step. Thermograms were analysed with software Netzsch Proteus Thermal Analysis 8.0.3 to identify *T*_g_ and calculate *C*_p_.

### Structural analysis

^29^Si and ^31^P magic angle spinning nuclear magnetic resonance (MAS NMR) spectra were acquired at 12.5 kHz using a Bruker Avance III 400 MHz NMR spectrometer at room temperature with an operating field strength of 9.4 T. Powder samples in a particle range of 38 to 125 μm were filled into a ZrO_2_ rotor 4 mm in diameter and spun at a spinning frequency of 12.5 kHz. The spectra are made up of an accumulation of 2048 and 96 scans for ^29^Si and ^31^P experiments, respectively, with a pulse angle of 90° and a recycle delay of 30 s between pulses. In ^29^Si MAS NMR, line broadening equal to 200 Hz was applied for the processing parameter. Data were collected and deconvoluted using the software Fityk; deconvolution into Gaussian functions was performed using as few Gaussian curves as necessary while trying to keep peak position and linewidth constant for the same Q^*n*^ species.^14^ Number of peaks and approximate peak positions were first determined for 45S5 based on available literature.^10,11^ Subsequently, peak position and fwhm obtained for 45S5 were used for fitting spectra of S53P4 and 13-93, while keeping peak position at ±4 ppm and fwhm at ±0.5 ppm.

Unpolarised Raman spectra were acquired using a Labram HR Evolution spectrometer equipped with a Peltier-cooled CCD and 1800 lines per mm grating. The samples were excited with a Coherent MX 488 nm solid-state laser focused through a 50× Olympus objective on the sample surface. The spectral resolution of the setup is about 1.7 cm^−1^, and the spatial resolution is about 1 μm. The laser power at the exit was adjusted to 60 mW on the sample. Spectra were acquired from 20 to 1500 cm^−1^, thanks to an ultra-low frequency filter used to attenuate the laser signal with an acquisition time of 120 s and an accumulation of 3 per spectral window. The collected Raman data were baseline corrected.

### Viscosity measurements

High viscosity measurements in the range of 10^8^ to 10^13^ Pa s were performed using a creep apparatus according to Neuville *et. al.*^26,34^ on rectangular glass samples (9 mm in height and around 3 mm in width and length). The temperature on top and bottom of the sample was recorded by two Pt–Pt/Rh10% thermocouples separately. A silver cylinder was fixed outside the measurement zone to ensure the temperature difference along the sample remained within 0.3 K throughout. The height change of the samples was measured during compression by means of two linear variable differential transformers located above and below the sample, and the deformation rate of sample was calculated with the height change as a function of time. Dynamic viscosity *η* (in Pa s) was subsequently calculated as described previously.^26,34^ The high viscosity measurement for one temperature was repeated more than four times under different applied stress. The calculated viscosity data of different stresses were averaged in the end to ensure accurate results.

Low viscosity measurements were performed in a vertical tube furnace as described by Neuville.^26^ This set-up can measure the viscosity of the glass in the temperature range from 1150 to 1950 K. A tailor-made cylindrical PtRh15% or PtIr10% crucible with a height of 50 mm and an inner diameter of 27 mm was fixed on an alumina tube leading from below to the interior of the furnace. After the glass in the crucible was heated to a molten state, a rotating cylinder possessing a diameter of 14 mm, a height of 21 mm and 23° conical extremities was submerged into the melt from the top of the crucible downwards.^26^ The viscosity was then calculated by the torque exerted on the rotating cylinder under the force of the melt at different angular velocities as described previously.^26^ At least five torques with different angular speeds were investigated for each temperature. The viscosity in the range of 10^4^ to 10^8^ Pa s could not be tested with the equipment used here, owing to crystallisation of the glasses studied.^34,35^

## Conclusions

We present structural analyses of the three most well-known bioactive glasses, 45S5 (Bioglass), S53P4 (BonAlive) and 13-93. While several published studies report on the structure of 45S5, the structure of the other two glasses, to the best of our knowledge, so far has not been analysed in detail. The three glasses not only differ in their composition, but this compositional variation results in differences in the degree of polymerisation of their silicate network, commonly referred to as network connectivity. In addition, glass 13-93 has a much higher alkaline earth metal oxide/alkali metal oxide ratio than the other two glasses. We show that both network connectivity and modifier cation field strength (*i.e.*, ratio of alkaline earth to alkali modifier ions) control the properties of bioactive glasses. An increase in network connectivity and cation field strength increases viscosity and transition temperature, resulting in a lower tendency to crystallise. On the other hand, this also reduces the tendency of the glasses to release ions and form apatite surface layers when in contact with aqueous solutions. However, as long as the differences in network connectivity are not too large, published literature suggests that differences in reactivity with water do not seem to result in pronounced differences in the glasses' *in vivo* bioactivity.

## Data availability

Data are available at the repository Zenodo: https://doi.org/10.5281/zenodo.14750536.

## Author contributions

Z Jin: formal analysis, investigation, writing – review & editing. DR Neuville: validation, formal analysis, writing – review & editing, funding acquisition. DS Brauer: conceptualization, methodology, validation, formal analysis, resources, writing – original draft, writing – review & editing, supervision, funding acquisition.

## Conflicts of interest

There are no conflicts to declare.
